# Topography induces differential sensitivity on cancer cell proliferation via Rho-ROCK-Myosin contractility

**DOI:** 10.1038/srep19672

**Published:** 2016-01-22

**Authors:** Parthiv Kant Chaudhuri, Catherine Qiurong Pan, Boon Chuan Low, Chwee Teck Lim

**Affiliations:** 1 Mechanobiology Institute, National University of Singapore, SINGAPORE; 2 Cell Signaling and Developmental Biology Laboratory, Department of Biological Sciences, National University of Singapore, SINGAPORE; 3 Department of Biomedical Engineering, National University of Singapore, SINGAPORE

## Abstract

Although the role of stiffness on proliferative response of cancer cells has been well studied, little is known about the effect of topographic cues in guiding cancer cell proliferation. Here, we examined the effect of topographic cues on cancer cell proliferation using micron scale topographic features and observed that anisotropic features like microgratings at specific dimension could reduce proliferation of non-cancer breast epithelial cells (MCF-10A) but not that for malignant breast cancer cells (MDA-MB-231 and MCF-7). However, isotropic features such as micropillars did not affect proliferation of MCF-10A, indicating that the anisotropic environmental cues are essential for this process. Interestingly, acto-myosin contraction inhibitory drugs, Y-27632 and blebbistatin prevented micrograting-mediated inhibition on proliferation. Here, we propose the concept of Mechanically-Induced Dormancy (MID) where topographic cues could activate Rho-ROCK-Myosin signaling to suppress non-cancerous cells proliferation whereas malignant cells are resistant to this inhibitory barrier and therefore continue uncontrolled proliferation.

Metastasis from a primary epithelial tumor is one of the major causes of cancer-related deaths. Cancer cells that are released from the primary tumor can eventually sow seeds for secondary metastatic tumors at distant sites^1^,^2^. Understanding how cancer cells establish these lesions is challenging. Various soluble components secreted by stromal cells of the metastatic niche are known to contribute to the specificity of the secondary location^3^. However, it is less understood whether the physical microenvironmental factors of the metastatic niche such as extracellular matrix (ECM) stiffness, dimensionality, and topography have any role in influencing the proliferation and colonization efficiency of the tumor cells.

The mammographic density in breast cancer patients is higher than healthy individuals due to increased collagen I cross-linking and the higher density is correlated with 4 to 6 times higher probability of developing breast cancer^4^,^5^,^6^. Higher collagen cross-linking promotes ECM stiffening, integrin clustering and focal adhesion formation that induce invasive responses in cancer cells^7^. On rigid ECMs, glioma cells spread rapidly with well defined stress fibers and the proliferation efficiency increases with higher ECM rigidity. However, inhibition of actomyosin contractility prevents this rigidity sensing and recovers the phenotypic changes thereby suggesting the involvement of non-muscle myosin-II based contractility in sensing ECM rigidity and promoting invasive phenotypes^8^. Interestingly on soft substrates, cells exert lesser contractile forces compared to rigid substrate but inhibiting actomyosin contraction promotes proliferation. This indicates that on compliant substrate, cellular contractility act as a barrier against proliferation^9^.

Apart from greater stiffness of the desmoplastic ECM, the architecture and organization of collagen fibers also undergo dynamic changes during tumor progression (tumor-associated collagen signature (TACS))^10^,^11^. Under normal conditions, the ECM fibers are arranged in a random, isotropic manner (TACS-1); however, during tumor growth the fibers appear in an organized and anisotropic arrangement (TACS-3)^12^. Malignant cells are contact guided by the clusters of linear collagen fibers and they use these aligned fibers as ‘highways’ to metastasize away from the primary tumor^13^. Aligned collagen matrices promote cellular adhesion along the fibers and provide minimal resistance to migration, thereby enhancing directional persistence and displacement^14^.

Pharmaceutical inhibitors against Rho-associated, coiled-coil containing protein kinase (ROCK) and myosin light chain kinase (MLCK) shows that migration of metastatic breast cancer cells, MDA-MB-231, along 3D collagen fiber is dependent on Rho- and ROCK-associated actomyosin contractility but not on MLCK signaling^15^. Recently, it was observed that in the presence of CXCL12 chemotactic gradient, the migration distance along aligned biomimetic nanofibers increased 82% for MDA-MB-231 cells; however, MCF-10A cells show insensitive response to the gradient^16^. Prostate cancer cells also preferentially migrate a greater distance along grooved topographies and the effect of topography is correlated with the metastatic potential of the cancer cells^17^. Although the above studies highlight the role of topographic cues on cancer cell migration, very little is known about the effect of topographic cues in influencing cancer cell proliferation. In one such study using lung carcinoma cells cultured on nano-featured surfaces, proliferation increased on 300 nm surfaces but decreased on 400 nm surfaces and apoptotic cells increased on 23 nm surfaces^18^. However, there was not much evidence of the mechanisms that could lead to these observations. Recently, Ortiz R. *et al*. studied the effect of micron scale gratings and square pits on the proliferative response of MDA-MB-231 and MCF-7 (non-metastatic breast cancer cells)^19^. However, they did not observe a significant difference in the proliferation of cancer cells in response to these topographies. It is plausible that the feature dimensions of these topographies ranged from 15 to 500 μm and cells might not be able to sense such larger size features (compared with cell size that is around 20 μm).

To delineate the role of topographic cues on cancer cell proliferation, we fabricated micron scale topographic features namely, gratings and pillars of different dimensions. We observed that microgratings provides anisotropic cues that reduced the proliferation of non-cancer breast epithelial cells (MCF-10A) but not of malignant breast cancer cells (MDA-MB-231 and MCF-7). Interestingly, actomyosin contraction inhibitory drugs, Y-27632 and blebbistatin, that blocks ROCK and non-muscle myosin-II, respectively, prevented the inhibitory effect of microgratings on non cancerous cell proliferation, thereby suggesting the involvement of non-muscle myosin-II based contractility in sensing topographic cues and reducing proliferation. We hereby propose the existence of a Mechanically-Induced Dormancy (MID) partly mediated by Rho-ROCK-Myosin pathway that could inhibit proliferation of normal cells but the cancerous cells could somehow bypass such growth inhibitory barrier and continue their unconstrained proliferation.

## Results and Discussion

### Microgratings reduces proliferation of non-cancer epithelial but not of metastatic breast cancer cells

The topographic features of the ECM underneath the basement membrane consist of a mixture of fibers, ridges and pores in the sub-micron scale^20^. In order to better mimic the topographic microenvironment *in-vivo*, we fabricated two basic micro-topographic features: gratings and pillars of different dimensions that have been previously shown to affect various cellular functions including proliferation^21^,^22^, differentiation^23^,^24^, and migration^17^,^25^ by controlling the distribution of cell surface receptors and cytoskeleton rearrangements. Gratings provide anisotropic cues to the cells thereby leading to preferential alignment in parallel direction to the grating axis^26^; however, pillars provide isotropic cues to the cells leading to random morphology^27^. To validate the reproducibility of our findings for a particular feature, we fabricated three different grating width of 2, 3 and 4 μm (Supplementary Table 1a), and three different diameters of micropillars at 2, 3 and 4 μm, (Supplementary Table 1b) respectively. A similar aspect ratio was maintained across the different diameters of the pillars to nullify the effect of rigidity change. The successful replication of different patterns was validated using scanning electron micrograph (SEM) as illustrated in Supplementary Fig. 1.

**Figure 1:**
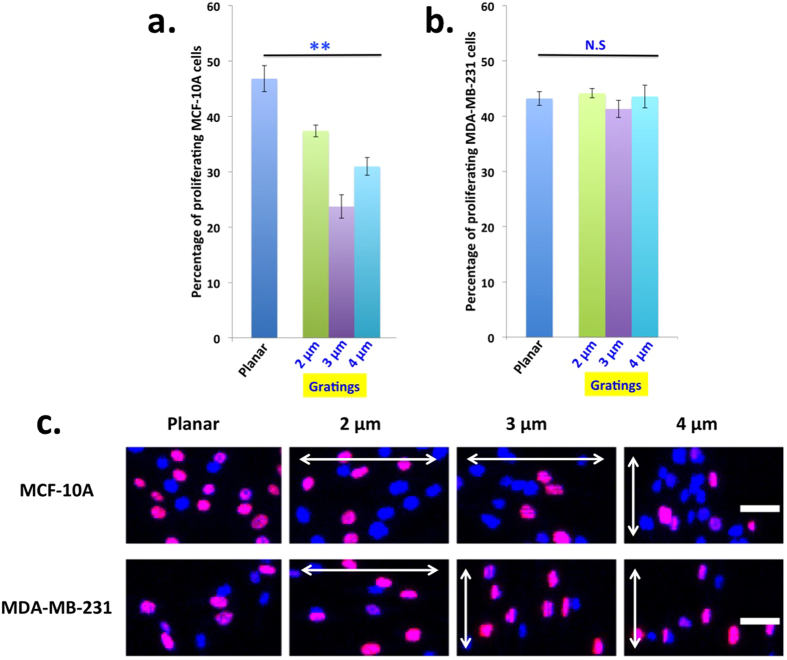
Microgratings reduces proliferation of non-cancer epithelial but not of metastatic breast cancer cells. (**a)** MCF-10A proliferation decreases on different dimensions of microgratings coated with collagen after 24 hours of cell seeding. Data are means ± s.e.m. (n = 3). For each experiment, 300 cells were considered on an average. P values were obtained using Student’s unpaired t-test. **p < 0.01 with respect to planar. (**b**) Topographical cues do not affect MDA-MB-231 proliferation. N.S. denotes non-significant with respect to planar. (**c**) Confocal microscopy representative images of proliferating MCF-10A and MDA-MB-231 cells cultured on different topographic patterns; DAPI: Blue, EdU: Red. Double-sided arrow indicates the direction of the grating axis. (Scale 50 μm)

To investigate the effect of topographical cues on non-cancer and cancer cell proliferation, MCF-10A and MDA-MB-231 cells were cultured for 24 hours on different gratings coated with collagen matrix. The collagen coating did not mask the topographic cues from the microgratings, as observed in previous report^28^. The proliferation percentage of MCF-10A decreased on different dimensions of microgratings when compared to the planar control (Fig. 1a). The greatest reduction in MCF-10A proliferation was observed for 3 μm gratings. It is believed that the 3 μm gratings may have provided an optimal dimension of the topographical cue for the cell surface receptors to sense in such a way as to reorganize the cellular cytoskeleton and reduce cell proliferation. To examine whether cell-cell adhesion or/and cell-matrix interaction plays a key role in reducing MCF-10A proliferation on microgratings, cells were seeded at lower density (Supplementary Fig. 2). Interestingly, we observed that microgratings reduced proliferation of MCF-10A even at lower cell density, thus highlighting the significance of cell-matrix interaction in the reduction in MCF-10A proliferation by microgratings. To determine if the microgratings induced reduction in MCF-10A proliferation is leading to apoptosis of the cells, we stained the cells for caspase activity and observed that there was no significant difference in apoptosis between cells grown on planar and microgratings (Supplementary Fig. 3). Therefore, microgratings induce MCF-10A cells to enter a dormant state after 24 hours of culture.

In contrast to MCF-10A cells, there was no significant change in the rate of proliferation in MDA-MB-231 across the different gratings, which indicates that topographical cues reduce proliferation of only the normal epithelial but not that of metastatic breast cancer cells (Fig. 1b). Confocal microscopy representative images of cells grown on different topographic dimensions were shown in Fig. 1c, where nucleus of all the cells was labeled with DAPI (indicated in blue) and proliferating nucleus was stained with EdU (indicated in red). Additionally, different pillar dimensions also did not affect the proliferation of MDA-MB-231 (Supplementary Fig. 4). Similarly, topographic features did not affect MCF-7 proliferation across different ECM proteins, namely, fibronectin, collagen and laminin after 24 hours of cell seeding (Supplementary Fig. 5). Therefore, the malignant cancer cells have some in-built mechanism to evade the proliferation inhibitory effect of topographical cues.

### Microgratings induces greatest reduction in proliferation of MCF-10A cells for collagen coating after 24 hours of cell seeding

**Figure 2:**
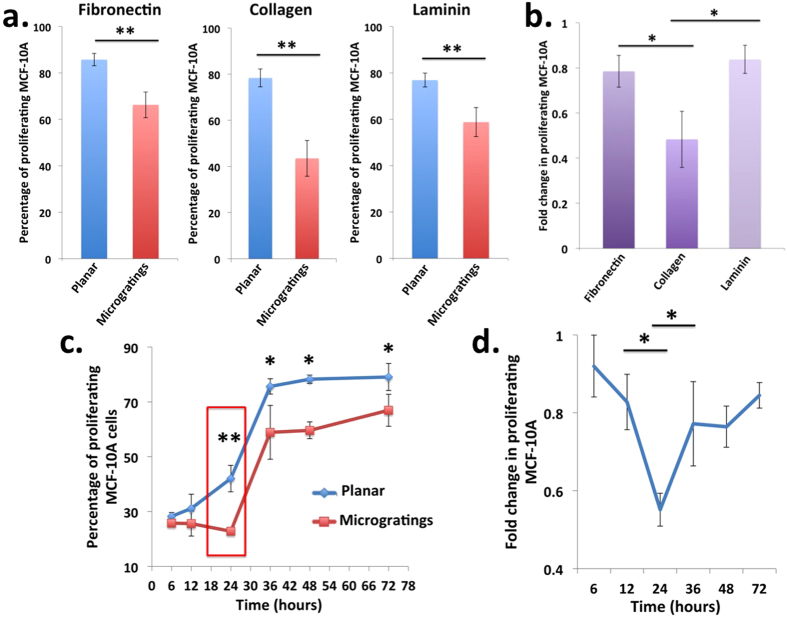
Microgratings induces greatest reduction in proliferation of MCF-10A cells for collagen coating after 24 hours of cell seeding. (**a)** MCF-10A proliferation was reduced by 2 μm microgratings across all the extracellular matrix (ECM) proteins, namely, fibronectin, collagen, laminin after 24 hours of cell seeding. Microgratings was coated with ECM proteins to facilitate cell adhesion, spreading and proliferation. (**b)** Greatest reduction in proliferation was observed for collagen coating. (**c)** Maximum reduction in proliferation occurred for collagen coating after 24 hours of cell seeding (indicated by red box). Blue and red colored line indicates the growth curve of cells seeded on planar and 2 μm microgratings respectively. Data are means ± s.e.m. (n = 3). For each experiment, 300 cells were considered on an average. P values were obtained using Student’s unpaired t-test. **p < 0.01 and *p < 0.05 with respect to planar. (**d**) Fold change calculation in proliferating cells as a function of time. *p < 0.05

To further characterize whether reduction in the micrograting-induced proliferation of MCF-10A is dependent on the specific cues from ECM, we seeded cells on microgratings coated with different ECM proteins namely, fibronectin, collagen and laminin, all of which are found to be present in the tumor micro-environment. Interestingly, the proliferation of MCF-10A decreases across all the three ECM proteins after 24 hours of cell seeding (Fig. 2a). This implies that topographical cues dominate over the biochemical cues provided by the different ECM matrices in reducing MCF-10A proliferation. The greatest reduction in proliferation of 0.48 fold was observed for collagen coating (Fig. 2b). Collagen is one of the most abundant ECM proteins in the cancer stroma and it contributes significantly to maintaining the tensile strength of the tissue^29^. Therefore, when cells are seeded on microgratings patterns, cell surface receptors might sense the specific chemical and physical cues associated with collagen matrix thereby leading to the greatest reduction in proliferation. Collagen-coated microgratings might trigger mechanotransduction signaling pathways involved in the reduction in cell proliferation either in an integrin dependent manner (through the activation of integrin receptor subunits β1, α1, α2, α10 or α11)^30^ or in an integrin independent manner (via the activation of Discoidin Domain Receptors (DDRs), a sub family of tyrosine kinase receptors)^31^. This might activate the tyrosine phosphorylation pathways involving Src and Focal Adhesion Kinases (FAK), which could lead to the reorganization of cytoskeletal tension and reduced proliferation.

Subsequently, we characterized the temporal dynamics of micrograting-induced dormancy of MCF-10A cells by culturing cells on collagen coated microgratings for different periods of time (Fig. 2c). Representative confocal microscopy images of the morphology for MCF-10A cells seeded on 2 μm microgratings at various time points are shown in Supplementary Fig. 6. The proliferation of cells on microgratings decreased at all time points starting from 12 hours to 72 hours after cell seeding. Therefore, cells need at least 12 hours to transduce the topographical cues from microgratings and thereafter reduce proliferation. Interestingly, the greatest reduction in proliferation of 0.5 fold was observed after 24 hours of cell seeding (Fig. 2d). The doubling time of MCF-10A is approximately 20 hours on normal tissue culture dishes^32^. Therefore, the proliferation inhibitory topographical cues might prevent the maximum division of cells in the M-phase of cell cycle and instead redirect the cells into G0 phase. However, the proliferation fold change tends to increase gradually over the subsequent time points from 36 to 72 hours, which suggests that cells tend to recover and develop resistance from the topography induced temporary dormancy with the passage of time. The mechanism underlying such long-term adaptation is now being investigated.

### Microgratings reduces MCF-10A proliferation through the activation of Rho-ROCK-Myosin pathway

**Figure 3:**
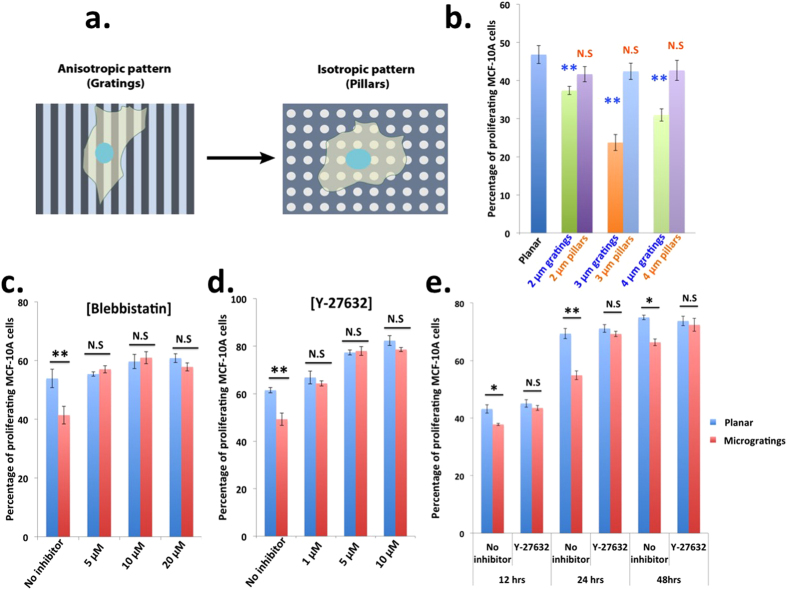
Microgratings reduces MCF-10A proliferation through the activation of Rho-ROCK-Myosin pathway. (**a)** Schematic representation of the morphology of cells seeded on anisotropic (gratings) and isotropic (pillars) patterns. (**b)** MCF-10A proliferation decreased on microgratings but not on micropillars of different diameters. Acto-myosin contractility plays a crucial role in the reduction of proliferation of MCF-10A cells on microgratings. Acto-Myosin contraction inhibitory drug treatment: (**c)** Blebbistatin (5 μM, 10 μM, 20 μM), (**d)** Y-27632 (1 μM, 5 μM, 10 μM), (**e)** Time course of cell proliferation after treatment with acto-myosin contraction inhibitory drug Y-27632 (5 μM) for various time points. Blue bar indicates planar and red indicates 2 μm microgratings. Data are means ± s.e.m. (n = 3). For each experiment, 300 cells were considered on an average. P values were obtained using Student’s unpaired t-test. **p < 0.01 with respect to planar. N.S. denotes non-significant difference compared to planar.

We then asked the question whether anisotropic cues provided by the microgratings is responsible for the reduction in MCF-10A proliferation. To answer this question, we sought to eliminate the anisotropy from microgratings by fabricating lines in perpendicular direction to the grating axis to create isotropic pillar patterns (Fig. 3a). MCF-10A was cultured for 24 hours on collagen-coated microgratings and micropillars of three different diameters, namely, 2, 3 and 4 μm as mentioned earlier. Interestingly, MCF-10A proliferation decreased on microgratings but not on micropillars of different topographic dimensions (Fig. 3b), signifying the importance of anisotropic cues for the induction of topography mediated temporary dormancy.

Next, we hypothesized that the topography-induced temporary dormancy of MCF-10A cells could be due to the generation of micrograting-mediated higher actomyosin contractility within the cell. To test this hypothesis, we treated MCF-10A cells cultured on microgratings with different concentrations of actomyosin contraction inhibitory drugs namely, blebbistatin (non muscle myosin II inhibitor) (5 μM, 10 μM, 20 μM) (Fig. 3c), Y-27632 (ROCK inhibitor) (1 μM, 5 μM, 10 μM) (Fig. 3d) and for different periods of time (12, 24, 48 hours) (Fig. 3e). Inhibition of actomyosin contraction prevented reduction in micrograting-mediated proliferation across the different concentrations and different time periods of the drug used. In other words, in the presence of actomyosin contractility inhibitory drugs, cells are unable to distinguish between planar and microgratings, which suggests the involvement of Rho-ROCK-Myosin based contractility in sensing topographic cues and reducing proliferation. Interestingly, cells plated on microgratings expressed less amount of myosin light chain (MLC) when compared to those cultured on the planar ones. However, they exhibited higher level of active and phosphorylated form of MLC when normalized to the total pool present in the cells (Supplementary Fig. 7). This result is consistent with the notion that despite reducing amount of myosin (probably owing to a yet unknown feedback mechanism), microgratings could activate Rho/ROCK/Myosin pathway. Our findings also support the previous report that microscale protrusions (‘micropegs’) reduced cell proliferation in fibroblasts and myoblasts through the generation of acto-myosin contractile forces^33^. Together, these studies highlight the significance of acto-myosin contractility-based signaling events in sensing mechanical cues from the cellular microenvironment and leading to the reduction in cell proliferation. However, it was unclear whether microscale protrusions lead to permanent or temporary reduction in fibroblasts and myoblasts cell proliferation. Our study therefore demonstrates the importance of anisotropic cues in the induction of topography-mediated reduction in cell proliferation that leads to temporary dormancy.

### Microgratings reduces spreading area in MCF-10A but not in MDA-MB-231 cells

**Figure 4:**
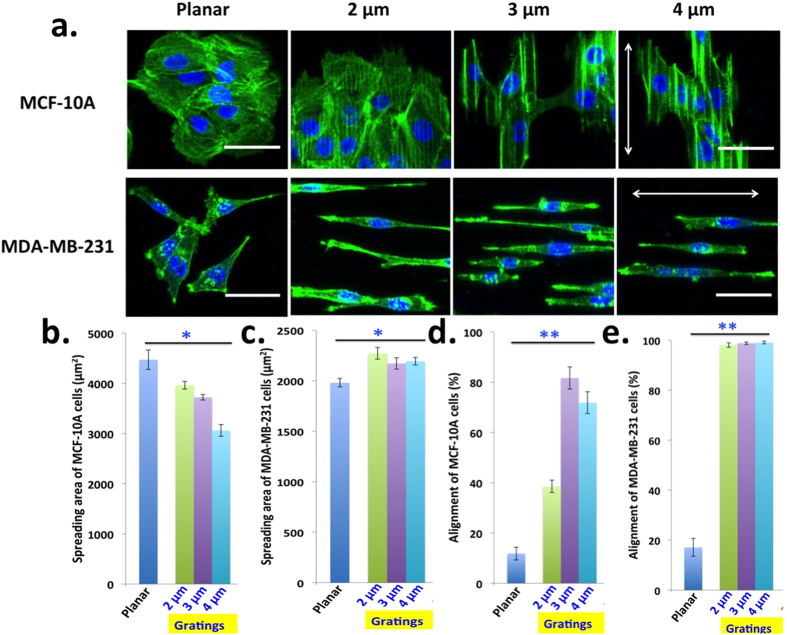
Microgratings reduces spreading area in MCF-10A but not in MDA-MB-231 cells. (**a)** Confocal microscopy representative images of the morphology of MCF-10A and MDA-MB-231 cells seeded on various topographic patterns. Double-sided arrow indicates the direction of the grating axis. (Phalloidin: Green; DAPI: Blue; Scale 50 μm). Spreading area of (**b)** MCF-10A cells and (**c)** MDA-MB-231 cells across various dimensions of microgratings. Spreading area decreases for MCF-10A but increases for MDA-MB-231 cells on microgratings. Alignment percentage of (**d)** MCF-10A cells and (**e)** MDA-MB-231 cells along the gratings. Both cells are aligned parallel to the microgratings. Data are means ± s.e.m. (n = 3). For each experiment, 200 cells were considered on an average. P values were obtained using Student’s unpaired t-test. **p < 0.01 and *p < 0.05 with respect to planar.

To examine whether the topography-induced temporary dormancy of MCF-10A is related to cell spreading, we cultured MCF-10A and MDA-MB-231 cells on collagen coated microgratings patterns for 24 hours and then stained for the F-actin cytoskeleton with phalloidin. Representative confocal microscopy images are shown in Fig. 4a. The spreading area of MCF-10A cells on microgratings decreased gradually with respect to the planar control as the grating width increased from 2 μm to 4 μm (Fig. 4b). Interestingly, microgratings did not significantly reduce MCF-10A cell spreading area after 24 hours treatment of acto-myosin contraction inhibitory drug Y-27632 (5 μM) (Supplementary Fig. 8). This indicates that microgratings induce activation of higher Rho-ROCK-Myosin based contractile forces that decrease MCF-10A cell spreading area and proliferation. However, microgratings did not reduce MCF-10A cell spreading area after 6 hours of cell seeding (Supplementary Fig. 9), consistent with previous indication that at least 12 hours are required for the transduction of topographical cues. In contrast, MDA-MB-231 cell spreading area increased on microgratings compared to planar surface (Fig. 4c). Higher invasive and metastatic potential of MDA-MB-231 cells might be responsible for this as they tend to follow the pathway of least resistance parallel to the grating where they could adhere and spread more, thereby resulting in more efficient and persistent migration, as was observed in a previous study^15^. Interestingly, both MCF-10A (Fig. 4d) and MDA-MB-231 (Fig. 4e) tend to align preferentially along the grating axis and MDA-MB-231 showed a remarkable higher alignment percentage of around 99% as evident from the confocal microscopy images as well and this finding is consistent with previous report^19^. We believe that parallel axis of the grating provides a more preferential path for cellular adhesion and migration along the gratings.

However, confocal microscopy images (Supplementary Fig. 10a) of cells grown on different diameters of micropillars indicate that micropillars reduced the spreading area for both MCF-10A (Supplementary Fig. 10b) and MDA-MB-231 (Supplementary Fig. 10c) cells. This is due to the limited area of cell attachment on top of the pillars whereas on a planar surface, there is a larger continuous surface for cell adhesion. Additionally, there was no preferential alignment of MCF-10A (Supplementary Fig. 10d) and MDA-MB-231 (Supplementary Fig. 10e) cells along any direction. This result is consistent with the notion that the micropillars provide an isotropic environment and as such, the cells do not have preference for any particular direction.

### Mechanically-Induced Dormancy (MID)

**Figure 5:**
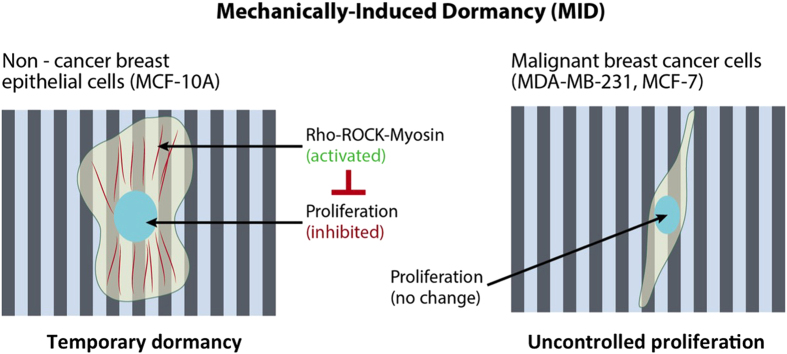
Schematic representation of Mechanically-Induced Dormancy (MID). The anisotropic topographical cues provided by microgratings could reduce the proliferation of MCF-10A cells and induce a temporary dormancy. However, MDA-MB-231 and MCF-7 cells could successfully overcome this temporary dormancy barrier and continue uncontrolled proliferation. Interestingly, treatment with acto-myosin contraction inhibitory drugs prevented topography induced temporary dormancy of MCF-10A, which suggests the involvement of Rho-ROCK-Myosin based contractility in sensing topographic cues and reducing proliferation.

In summary, we have unraveled a previously unknown proliferation inhibitory effect of mechanical cues (Mechanically-Induced Dormancy; MID) that exerts temporary dormancy in the normal epithelial cells but somehow the malignant cells could overcome such barrier with unaffected proliferation (Fig. 5). The desmoplastic ECM is dynamically remodeled in a highly organized and anisotropic manner by the activation of stromal cell in the tumor microenvironment^12^. The cancer cells utilize this anisotropy to their own advantage by using the aligned collagen fibers as ‘highways’ to migrate away from the primary tumor in a more efficient and persistent manner^16^. But it was not known previously what role this anisotropic environment has on the proliferation efficiency of the cancer cells. We mimicked this anisotropic topography *in-vitro* by fabricating microgratings of different dimensions using micro-fabrication. We observed that the anisotropic topographical cues could reduce the proliferation of MCF-10A and induce a temporary dormancy. However, MDA-MB-231 and MCF-7 cells could successfully overcome this temporary dormancy barrier. Interestingly, treatment with Y-27632 and blebbistatin prevented topography induced temporary dormancy of MCF-10A, which suggests the involvement of Rho-ROCK-Myosin based contractility in sensing topographic cues and reducing proliferation. The mechanism by which Rho-ROCK-Myosin senses these unique cues and how cancer cells bypass this inhibitory barrier is now being investigated. This study highlights the importance of mechanical (topographical) cues in maintaining normal tissue homeostasis during healthy conditions. However, during a diseased condition e.g. (cancer outgrowth), this proliferation inhibitory mechanical cue fails to provide a barrier and might be one of the contributing factors for the uncontrolled proliferation of cancer cells. In future, it will be interesting to mimic these topographic cues under physiologically relevant *in vivo* conditions and also in 3D cell culture *in vitro* systems to obtain further insight into this exciting phenomenon of growth control.

A better mechanistic understanding of how the presence of myriads of physical cues in the proliferative and metastatic niche contributes to the progress of tumorigenesis and metastasis will lead to the development of mechanobiologically-inspired anti-cancer treatments. Promising chemotherapeutic agents targeting cellular contractility and adhesion machineries including the integrin inhibitor Cilengitide^34^ and the low molecular weight FAK (focal adhesion kinase) antagonist TAE226^35^, has already started to emerge, and more are expected to come.

## Methods

### Fabrication of topographic patterns

Different topographic patterns were fabricated using SU-8 on silicon wafers. The dimensions of each pattern were 1 × 1 cm. The surrounding unpatterned region outside the topographic patterns was used as planar control. Curing agent : polydimethylsiloxane (PDMS; Sylgard 184, Dow Corning) of 1 : 10 ratio was mixed homogenously and poured on silanized silicon wafer. Degassing was done to remove all air bubbles formed during mixing. After degassing, PDMS was cured for 2 hours at 80 °C. Cured PDMS was peeled off from the silicon wafer and subsequently used for cell seeding. To validate the successful replication of different topographic patterns, PDMS substrates were sputter-coated with 11-nm-thick platinum (JEOL JFC 1600 Auto Fine Coater) and imaged with field emission scanning electron microscope (SEM) (JEOL).

### Cell culture

Non-cancer human breast epithelial cells (MCF-10A; American Type Culture Collection (ATCC)) were cultured in mammary epithelial growth medium (MEGM Bullet Kit; Lonza Corporation), supplemented with cholera toxin (100 ng mL^−1^). Prior to cell seeding on topographic patterns, cells were synchronized in the G0/G1 phase by growing cells to 100% confluence for 1 to 3 days in the same medium^36^. Synchronized cells were then trypsinized and seeded on the patterns. The culture of human metastatic breast cancer cell line (MDA-MB-231; ATCC) and human non-metastatic breast cancer cell line (MCF-7; ATCC) was carried out in DMEM supplemented with 10% fetal bovine serum, 1% penicillin and streptomycin at 37 °C in 5% CO_2_ environment. Cells were trypsinised on reaching 80% confluence. Prior to seeding on the patterns, the cells were serum starved overnight to synchronize the cell cycle.

The PDMS patterns were cleaned, air-plasma treated for 3 minutes (Model PDC-002, Harrick Scientific Corp) and sterilized with 70% isopropanol and UV for 5 minutes. They were subsequently coated with 10 μg ml^−1^ fibronectin (Sigma-Aldrich), 20 μg ml^−1^ collagen I (Bovine; Nutragen) or 20 μg ml^−1^ laminin (from Engelbreth–Holm–Swarm murine sarcoma; Invitrogen) for 1 hour at 37 °C. Excess protein was then washed away prior to cell seeding. 70,000 cells per pattern were seeded to maintain 70% confluence. The cells were cultured on the patterns for different periods of time ranging from 6 hours to 72 hours, as mentioned in the text.

### Cell proliferation and apoptosis assay

After culturing the cells on the patterns, the cells were washed with 1X phosphate buffered saline (PBS, Sigma Aldrich) and subsequently fixed with 4% (w∕v) paraformaldehyde (PFA, Sigma Aldrich) for 20 minutes. 0.01% Triton X‐100 was used for 10 minutes to permeabilize the cells. Proliferating cells were identified using Edu staining from Click-iT™ EdU Alexa Fluor® 555 Imaging Kit (Invitrogen, C10338). The nucleus of the cells was labeled with DAPI stain (Invitrogen, D1306) and were imaged using Nikon Confocal A1R microscope. Proliferating cells (stained with EdU) and total number of cells (stained with DAPI) were manually counted and ratio between them was done to obtain the percentage of proliferating cells. For each experiment, 300 cells were considered on an average. In order to identify apoptotic cells, cells were cultured in presence of 2 μM NucView^TM^ 530 Caspase-3 Substrate for 24 hours and then washed with 1X PBS and fixed using 4% PFA. The nucleus of the cells was stained with DAPI and fluorescent microscopy was performed.

### Inhibitor drug treatment

To perform drug inhibition experiments, different concentrations (5 μM, 10 μM, 20 μM) of Blebbistatin (non-muscle Myosin II inhibitor; Sigma-Aldrich) and different concentrations (1 μM, 5 μM, 10 μM) of Y-27632 (ROCK inhibitor; Sigma-Aldrich) were added to the cell culture media 6 hours after cell seeding, to allow the cells to adhere and spread properly. The cells were cultured in the presence of inhibitory drugs for 18 hours and the media was replenished with fresh media containing the same concentration of drug once after 9 hours, to avoid any confounding effect due to inactivation of the drug during the long incubation time period.

### Quantification of cell spreading and alignment

Samples were fixed using 4% PFA and permeabilized with 0.01% Triton X‐100. F-actin was stained using Green 488 phalloidin (Invitrogen) and the nucleus was labeled with DAPI. Samples were washed thrice with 1X PBS and imaged using Nikon Confocal A1R microscope. Cell boundary was traced manually using ImageJ to measure cell area and angle. Alignment of cells was considered if the angle between the major axis of the cell and the grating was less than 15°. The alignment percentage of the cells was determined and for each experiment, an average of 200 cells were considered.

### Western blot analyses

Cell lysates were separated by gradient SDS-PAGE gels (Biorad) followed by western blotting with 1 hour of blocking (5% BSA in 0.1% Tween in TBS) at room temperature (RT), overnight primary antibody incubation at 4 °C, and three washes of 10 minutes each at RT. Blots were then incubated with secondary antibody for 1 hour at RT and final three washes of 10 minutes each at RT. Immunoblots were developed using Pierce Pico ECL (Thermo Scientific). Antibodies used were polyclonal phospho-MLC of Thr18/Ser19 (CST), polyclonal MLC (CST) and monoclonal α-tubulin (Sigma).

### Statistics

Student’s unpaired t-test was used for statistical analysis. Each experiment was repeated at least thrice. Data represents ± s.e.m (standard error of mean) for independent experiments (n = 3). Statistical significance was considered at *p* < 0.05 and *p* < 0.01. All statistical comparisons were based on experiments performed on the same day.

## Additional Information

**How to cite this article**: Chaudhuri, P.K. *et al*. Topography induces differential sensitivity on cancer cell proliferation via Rho-ROCK-Myosin contractility. *Sci. Rep.* **6**, 19672; doi: 10.1038/srep19672 (2016).

## Supplementary Material

Supplementary Information

## References

[b1] Gupta G. P. & Massagué J. Cancer metastasis: building a framework. Cell. 127, 679–695 (2006). 17110329 10.1016/j.cell.2006.11.001

[b2] Nguyen D. X., Bos P. D. & Massagué J. Metastasis: from dissemination to organ-specific colonization. Nat Rev Cancer. 9, 274–284 (2009). 19308067 10.1038/nrc2622

[b3] Müller A. . Involvement of chemokine receptors in breast cancer metastasis. Nature. 410, 50–56 (2001). 11242036 10.1038/35065016

[b4] Wolfe J. N. Risk for breast cancer development determined by mammographic parenchymal pattern. *Cancer*. 37, 2486–2492 (1976). 1260729 10.1002/1097-0142(197605)37:5<2486::aid-cncr2820370542>3.0.co;2-8

[b5] Boyd N. F. . Mammographic density and the risk and detection of breast cancer. N Engl J Med. 356, 227–236 (2007). 17229950 10.1056/NEJMoa062790

[b6] Boyd N. F., Lockwood G. A., Byng J. W., Tritchler D. L. & Yaffe M. J. Mammographic densities and breast cancer risk. Cancer Epidemiol Biomarkers Prev. 7, 1133–1144 (1998). 9865433

[b7] Levental K. R. . Matrix crosslinking forces tumor progression by enhancing integrin signaling. Cell. 139, 891–906 (2009). 19931152 10.1016/j.cell.2009.10.027 PMC2788004

[b8] Ulrich T. A., de Juan Pardo E. M. & Kumar S. The mechanical rigidity of the extracellular matrix regulates the structure, motility, and proliferation of glioma cells. Cancer Res. 69, 4167–4174 (2009). 19435897 10.1158/0008-5472.CAN-08-4859 PMC2727355

[b9] Mih J. D., Marinkovic A., Liu F., Sharif A. S. & Tschumperlin D. J. Matrix stiffness reverses the effect of actomyosin tension on cell proliferation. J. Cell Sci. 125, 5974–5983 (2012). 23097048 10.1242/jcs.108886 PMC3585515

[b10] Conklin M. W. . Aligned collagen is a prognostic signature for survival in human breast carcinoma. Am J Pathol. 178, 1221–1232 (2011). 21356373 10.1016/j.ajpath.2010.11.076 PMC3070581

[b11] Provenzano P. P. . Collagen reorganization at the tumor-stromal interface facilitates local invasion. BMC Med. 4, 38 (2006). 17190588 10.1186/1741-7015-4-38 PMC1781458

[b12] Goetz J. G. . Biomechanical remodeling of the microenvironment by stromal caveolin-1 favors tumor invasion and metastasis. Cell. 146, 148–163 (2011). 21729786 10.1016/j.cell.2011.05.040 PMC3244213

[b13] Ingman W. V., Wyckoff J., Gouon-Evans V., Condeelis J. & Pollard J. W. Macrophages promote collagen fibrillogenesis around terminal end buds of the developing mammary gland. Dev Dyn. 235, 3222–3229 (2006). 17029292 10.1002/dvdy.20972

[b14] Friedl P. & Wolf K. Tube travel: the role of proteases in individual and collective cancer cell invasion. Cancer Res. 68, 7247–7249 (2008). 18794108 10.1158/0008-5472.CAN-08-0784

[b15] Riching K. M. . 3D Collagen Alignment Limits Protrusions to Enhance Breast Cancer Cell Persistence. Biophys J. 107, 2546–2558 (2014). 25468334 10.1016/j.bpj.2014.10.035 PMC4255204

[b16] Nelson M. T. . Preferential, enhanced breast cancer cell migration on biomimetic electrospun nanofiber ‘cell highways’. BMC Cancer. 14, 825 (2014). 25385001 10.1186/1471-2407-14-825 PMC4236463

[b17] Sarna M., Wybieralska E., Miekus K., Drukala J. & Madeja Z. Topographical control of prostate cancer cell migration. Mol Med Rep . 2, 865–871 (2009). 21475914 10.3892/mmr_00000185

[b18] Zhang L. & Webster T. J. Decreased lung carcinoma cell functions on select polymer nanometer surface features. J. Biomed Mater Res A . 100, 94–102 (2012). 21987490 10.1002/jbm.a.33217

[b19] Ortiz R. . Ultra-fast laser microprocessing of medical polymers for cell engineering applications. Mater Sci Eng C Mater Biol Appl . 37, 241–250 (2014). 24582245 10.1016/j.msec.2013.12.039

[b20] Flemming R. G., Murphy C. J., Abrams G. A., Goodman S. L. & Nealey P. F. Effects of synthetic micro-and nano-structured surfaces on cell behavior. Biomaterials. 20, 573–588 (1999). 10213360 10.1016/s0142-9612(98)00209-9

[b21] Den Braber E. T. . Quantitative analysis of cell proliferation and orientation on substrata with uniform parallel surface micro-grooves. Biomaterials. 17, 1093–1099 (1996). 8718969 10.1016/0142-9612(96)85910-2

[b22] Rebollar E. . Proliferation of aligned mammalian cells on laser-nanostructured polystyrene. Biomaterials. 29, 1796–1806 (2008). 18237776 10.1016/j.biomaterials.2007.12.039

[b23] Yim E. K., Pang S. W. & Leong K. W. Synthetic nanostructures inducing differentiation of human mesenchymal stem cells into neuronal lineage. Exp Cell Res. 313, 1820–1829 (2007). 17428465 10.1016/j.yexcr.2007.02.031 PMC2038987

[b24] Gerecht S. . The effect of actin disrupting agents on contact guidance of human embryonic stem cells. Biomaterials. 28, 4068–4077 (2007). 17576011 10.1016/j.biomaterials.2007.05.027 PMC2257875

[b25] Yim E. K. . Nanopattern-induced changes in morphology and motility of smooth muscle cells. Biomaterials. 26, 5405–5413 (2005). 15814139 10.1016/j.biomaterials.2005.01.058 PMC2376810

[b26] Oakley C. & Brunette D. M. The sequence of alignment of microtubules, focal contacts and actin filaments in fibroblasts spreading on smooth and grooved titanium substrata. J. Cell Sci. 106, 343–354 (1993). 8270636 10.1242/jcs.106.1.343

[b27] Moe A. A. . Microarray with Micro‐and Nano‐topographies Enables Identification of the Optimal Topography for Directing the Differentiation of Primary Murine Neural Progenitor Cells. Small. 8, 3050–3061 (2012). 22807278 10.1002/smll.201200490

[b28] Yim E. K. . Nanopattern-induced changes in morphology and motility of smooth muscle cells. Biomaterials. 26, 5405–13 (2005). 15814139 10.1016/j.biomaterials.2005.01.058 PMC2376810

[b29] Kolácná L. . Biochemical and biophysical aspects of collagen nanostructure in the extracellular matrix. Physiol Res. 56, 51–60 (2007). 17552894 10.33549/physiolres.931302

[b30] Teo B. K. . Nanotopography modulates mechanotransduction of stem cells and induces differentiation through focal adhesion kinase. ACS Nano. 7, 4785–98 (2013). 23672596 10.1021/nn304966z

[b31] Vogel W. F., Abdulhussein R. & Ford C. E. Sensing extracellular matrix: an update on discoidin domain receptor function. Cell Signal. 18, 1108–16 (2006). 16626936 10.1016/j.cellsig.2006.02.012

[b32] Starcevic S. L., Diotte N. M., Zukowski K. L., Cameron M. J. & Novak R. F. Oxidative DNA damage and repair in a cell lineage model of human proliferative breast disease (PBD). Toxicol Sci. 75, 74–81 (2003). 12805649 10.1093/toxsci/kfg154

[b33] Thakar R. G. . Contractility-dependent modulation of cell proliferation and adhesion by microscale topographical cues. Small. 4, 1416–24 (2008). 18711756 10.1002/smll.200701302

[b34] Reardon D. A., Nabors L. B., Stupp R. & Mikkelsen T. Cilengitide: an integrin-targeting arginine-glycine-aspartic acid peptide with promising activity for glioblastoma multiforme. Expert Opin Investig Drugs. 17, 1225–35 (2008). 10.1517/13543784.17.8.1225 PMC2832832 18616418

[b35] Shi Q. . A novel low‐molecular weight inhibitor of focal adhesion kinase, TAE226, inhibits glioma growth. Mol Carcinog. 46, 488–496 (2007). 17219439 10.1002/mc.20297

[b36] Gajewski E. . Oxidative DNA base damage in MCF-10A breast epithelial cells at clinically achievable concentrations of doxorubicin. Biochem Pharmacol. 73, 1947–1956 (2007). 17445777 10.1016/j.bcp.2007.03.022 PMC2693330

