# Supplementary material for: Topography induces differential sensitivity on cancer cell proliferation via Rho-ROCK-Myosin contractility
Source: Sci Rep. 2016 Jan 22;6:19672. doi: 10.1038/srep19672 (PMC4726280; doi:10.1038/srep19672)
Supplement: Supplementary Information [file srep19672-s1.pdf]

Topography induces differential sensitivity on cancer cell proliferation via Rho-ROCK-Myosin contractility

Parthiv Kant Chaudhuri[1], Catherine Qiurong Pan[1], Boon Chuan Low[1,2#], Chwee Teck Lim[1,3#]

Supplementary figures

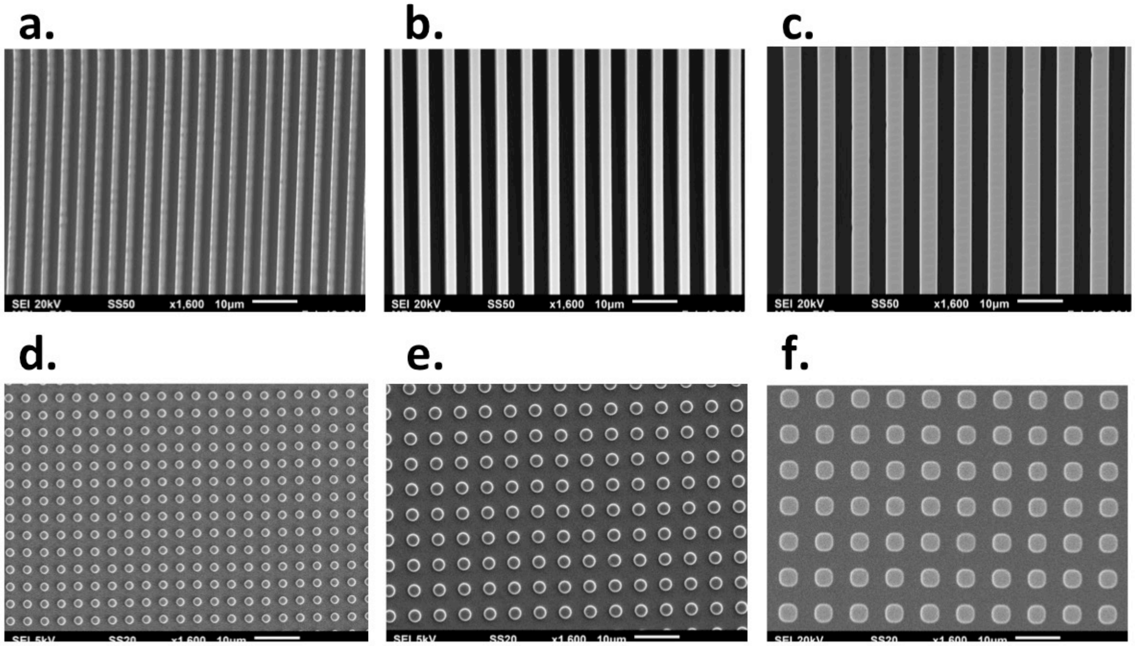

Supplementary Figure 1: Scanning electron microscopy (SEM) of different topographic patterns. Gratings: **a.** 2 μm, **b.** 3 μm, **c.** 4 μm; Pillars: **d.** 2 μm, **e.** 3 μm, **f.** 4 μm (Scale 10 μm).

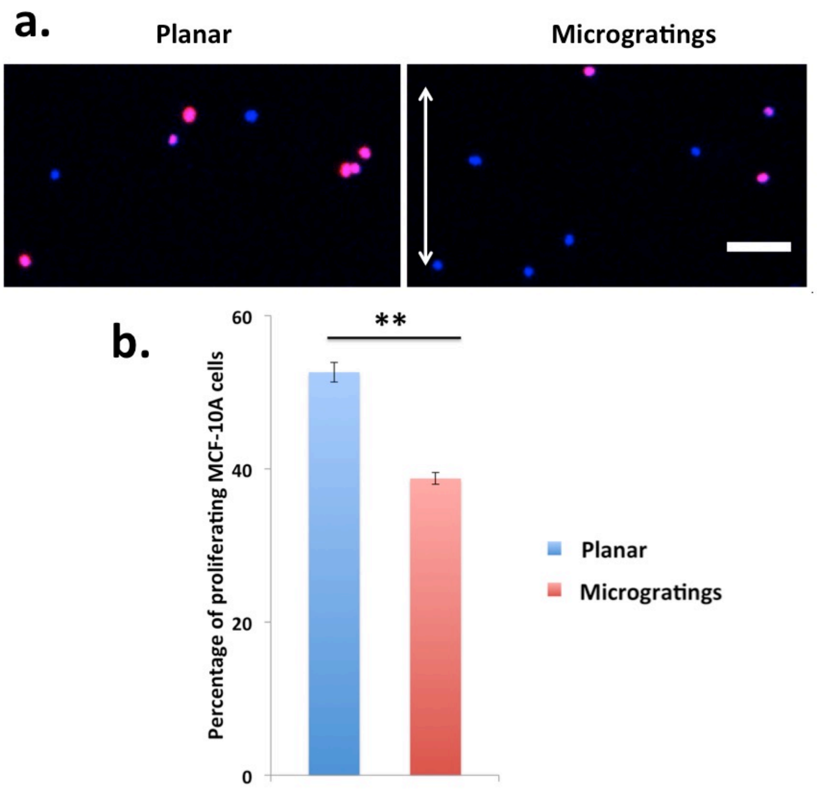

Supplementary Figure 2: Microgratings reduces MCF-10A proliferation at low density.

a. Confocal microscopy representative images of proliferating MCF-10A cells cultured on 2 μm microgratings; DAPI: Blue, EdU: Red (Scale 100 μm). Double-sided arrow indicates the direction of the grating axis. **b.** Percentage of proliferating MCF-10A cells. Data are means ±s.e.m. (n=3). For each experiment, 300 cells were considered on an average. P values were obtained using Student's unpaired t-test. ** $p < 0.01$ with respect to planar.

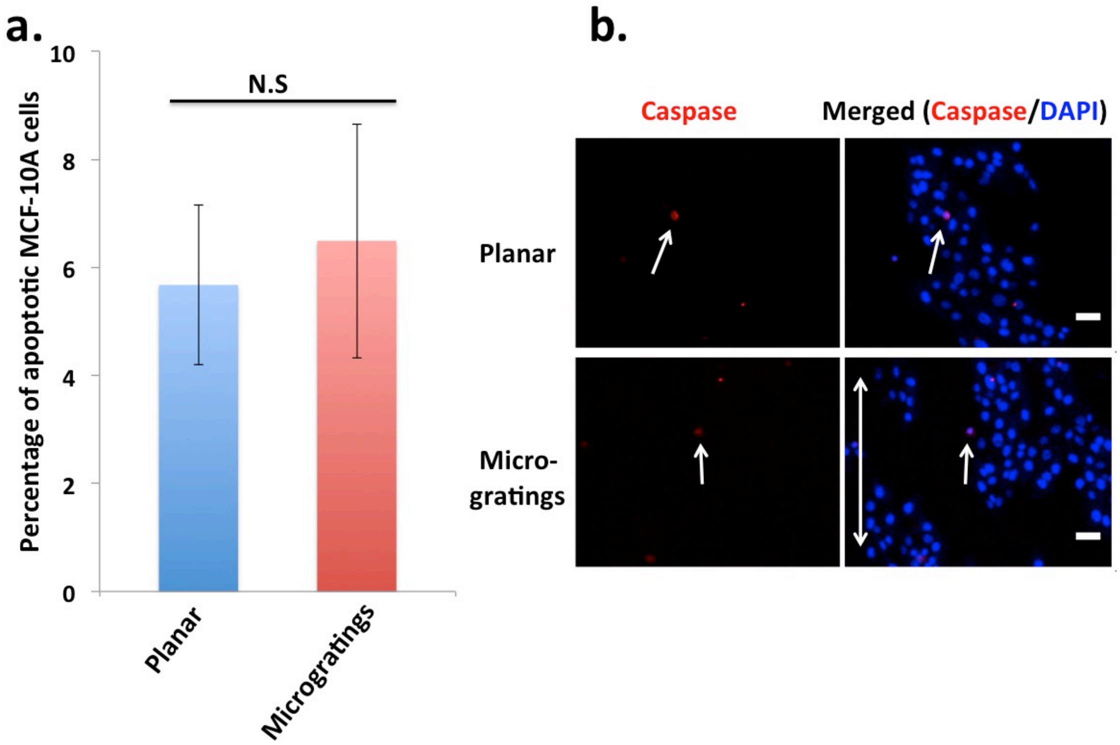

Supplementary Figure 3: Microgratings do not lead to apoptosis of MCF-10A cells.

a. Percentage of apoptotic MCF-10A cells. No significant difference in apoptosis between cells grown on planar and 2 μm microgratings. Data are means ±s.e.m. (n=3). For each experiment, 200 cells were considered on an average. N.S. denotes non-significant difference compared to planar. **b.** Confocal microscopy representative images of apoptotic cells (arrow). Double-sided arrow indicates the direction of the grating axis. (Scale 50 μm)

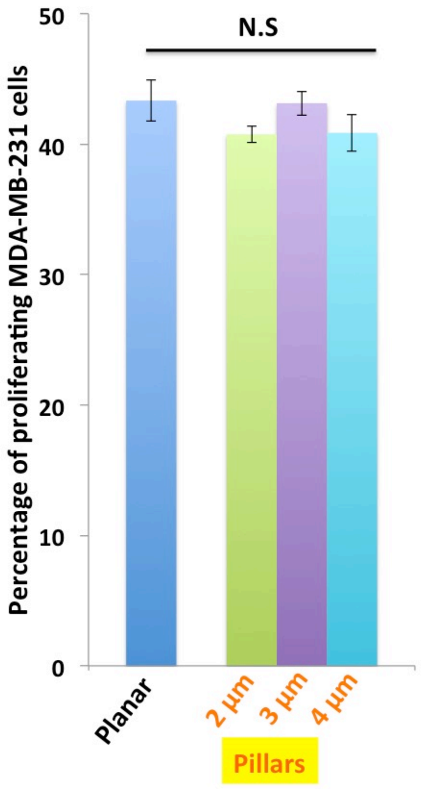

Supplementary Figure 4: Different pillar diameters do not affect MDA-MB-231 cell proliferation. Data are means ±s.e.m. (n=3). For each experiment, 300 cells were considered on an average. N.S. denotes non-significant difference compared to planar.

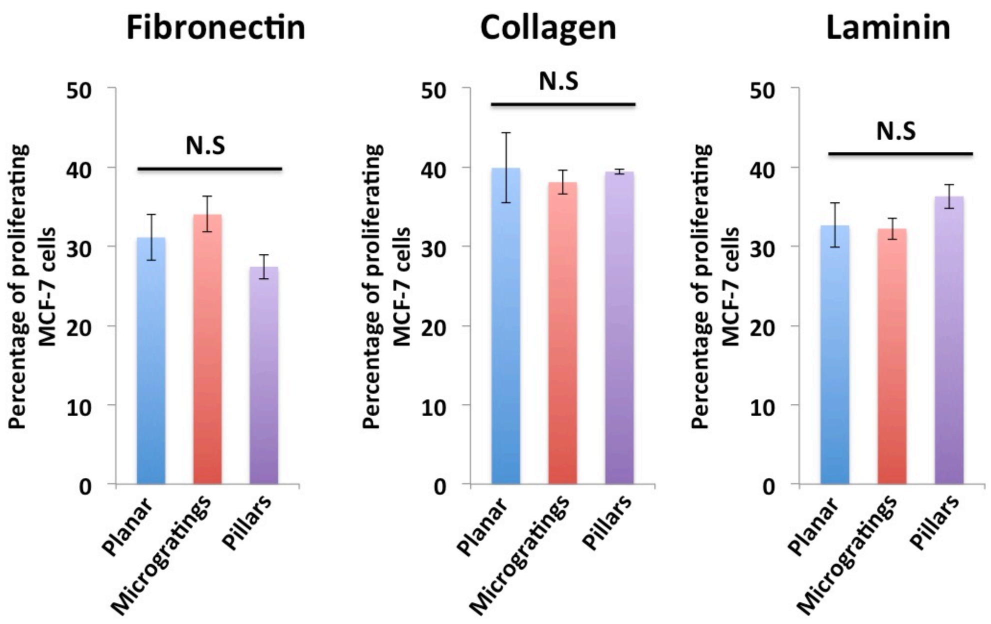

Supplementary Figure 5: Topographic features do not affect non-metastatic breast cancer cell (MCF-7) proliferation across different ECM proteins after 24 hours of cell seeding. Cells were seeded on 2 μm microgratings and 2 μm micropillars. Data are means ±s.e.m. (n=3). For each experiment, 300 cells were considered on an average. N.S. denotes non-significant difference compared to planar.

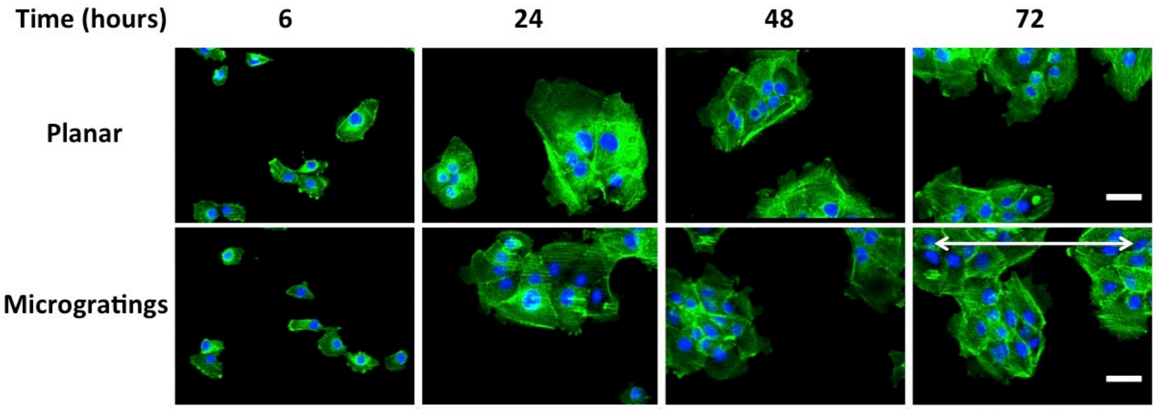

Supplementary Figure 6: Confocal microscopy representative images of the morphology of MCF-10A cells seeded on 2 µm microgratings for various time points. Double-sided arrow indicates the direction of the grating axis (Phalloidin: Green; DAPI: Blue; Scale 50 µm).

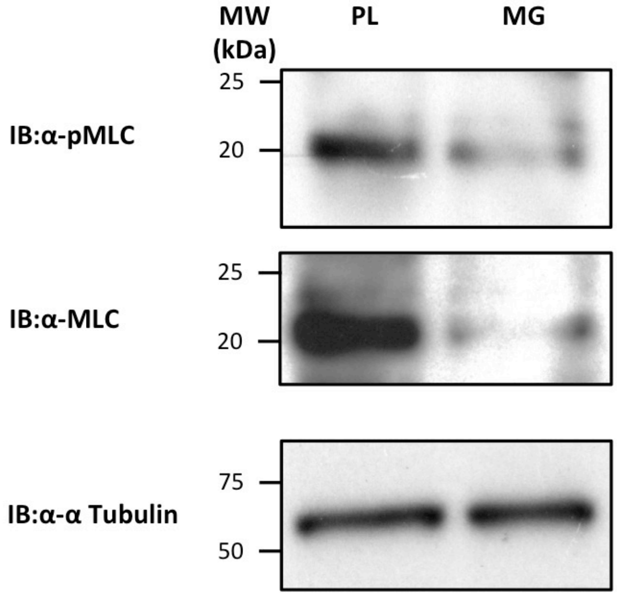

Supplementary Figure 7: Lysates from MCF-10A cells grown on planar (PL) or microgratings (MG) were separated by gradient gel and immunoblotted (IB) with antibodies for phospho-MLC (pMLC), MLC and α-tubulin as loading control.

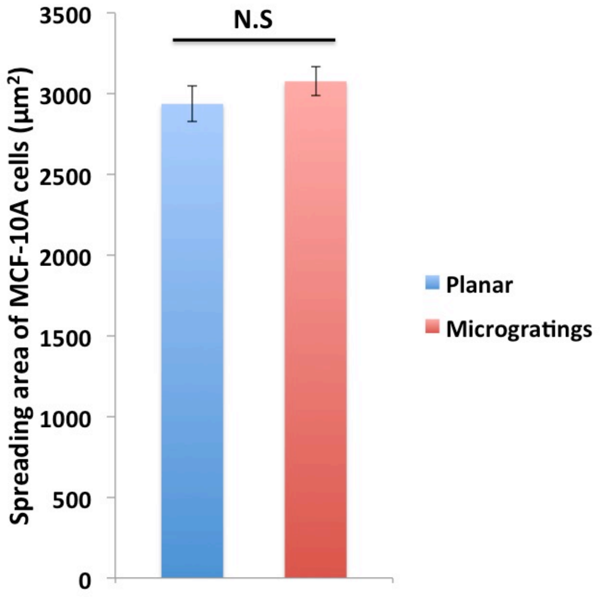

Supplementary Figure 8: Microgratings does not reduce MCF-10A cell spreading area after 24 hours treatment of acto-myosin contraction inhibitory drug Y-27632 (5 µM). Data are means ±s.e.m. (n=3). For each experiment, 200 cells were considered on an average. N.S. denotes non-significant difference compared to planar.

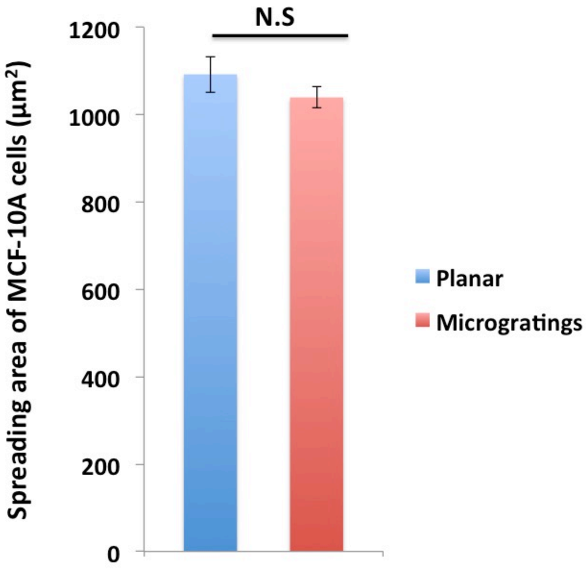

Supplementary Figure 9: Microgratings does not reduce MCF-10A cell spreading area after 6 hours of cell seeding. Data are means ±s.e.m. (n=3). For each experiment, 200 cells were considered on an average. N.S. denotes non-significant difference compared to planar.

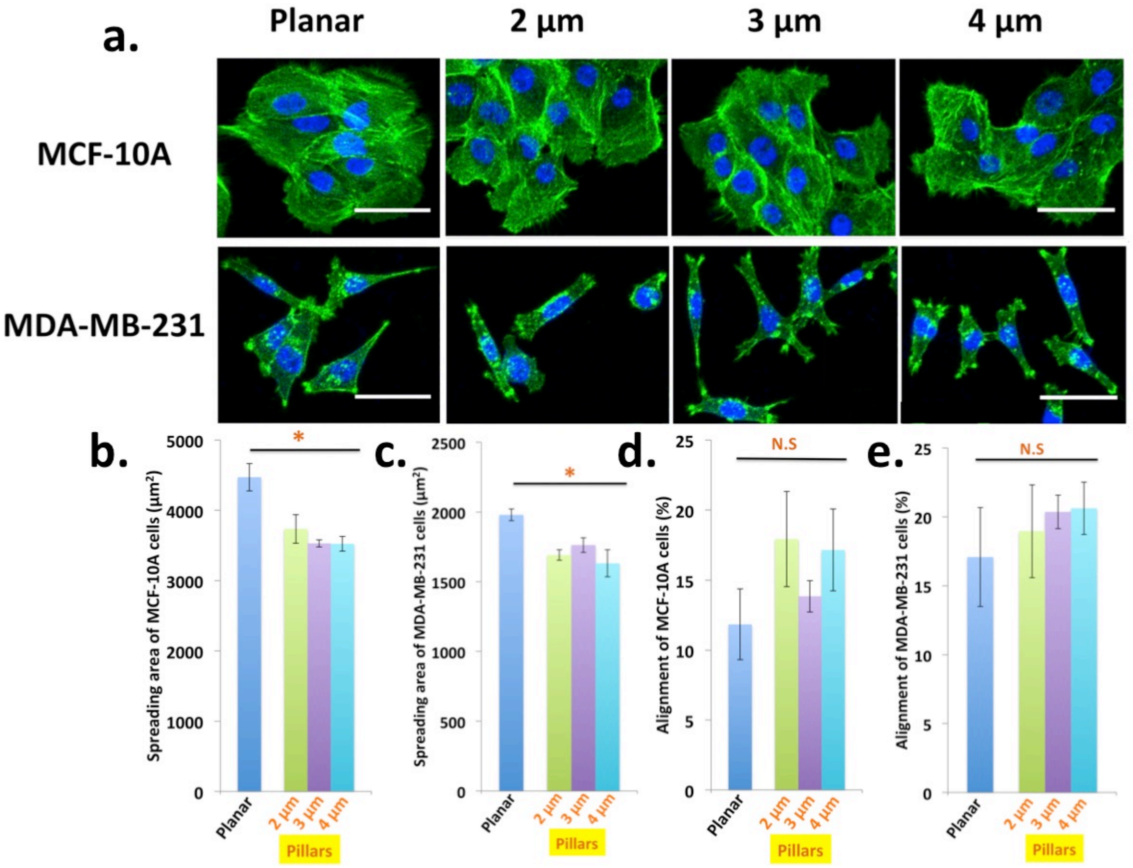

Supplementary Figure 10: Micropillars reduces spreading area of both MCF-10A and MDA-MB-231 cells.

a. Confocal microscopy representative images of the morphology of MCF-10A and MDA-MB-231 cells seeded on various topographic patterns. (Phalloidin: Green; DAPI: Blue; Scale 50 μm). Spreading area of **b.** MCF-10A cells and **c.** MDA-MB-231 cells across various diameters of micropillars. Spreading area decreases for both MCF-10A and MDA-MB-231 cells. Alignment percentage of **d.** MCF-10A cells and **e.** MDA-MB-231 cells along the pillars. No significant alignment of cells was observed. Data are means ±s.e.m. (n=3). For each experiment, 200 cells were considered on an average. P values were obtained using Student's unpaired t-test. * $p < 0.05$ with respect to planar and N.S. denotes non-significant difference compared to planar.

Supplementary Table 1a: Dimension of grating patterns

No.	Width (µm)	Space (µm)	Height (µm)
**1**	2	2	8
**2**	3	3	8
**3**	4	4	8

Supplementary Table 1b: Dimension of pillar patterns

No.	Pillar diameter (µm)	Centre to Centre distance (µm)	Height (µm)
**1**	2	4	2
**2**	3	6	2
**3**	4	8	2
